# Short-Term Practice Modulates ERP Components Without Behavioral Change in a Short-ISI Go/NoGo Task

**DOI:** 10.3390/brainsci15111208

**Published:** 2025-11-09

**Authors:** Yasushi Sugawara, Yuya Matsuda, Ryo Kurokawa, Rin Kosuge, Satoshi Kudoh, Mayu Akaiwa, Hidekazu Saito, Takeshi Sasaki, Kazuhiro Sugawara

**Affiliations:** 1 Department of Rehabilitation, Sapporo Hakuyokai Hospital, Sapporo 062-0931, Japan; yasushi.sug@gmail.com (Y.S.);; 2 Graduate School of Health Sciences, Sapporo Medical University, Sapporo 060-8556, Japan; 3 Department of Human Health Sciences, Graduate School of Medicine, Kyoto University, Kyoto 606-8501, Japan; 4 Department of Occupational Therapy, School of Health Sciences, Sapporo Medical University, Sapporo 060-8556, Japan; 5 Department of Physical Therapy, School of Health Sciences, Sapporo Medical University, Sapporo 060-8556, Japan

**Keywords:** event-related potentials, visual motor task, inter-stimulus interval, repeated training, response inhibition task

## Abstract

Background/Objectives: Response inhibition, a core aspect of executive function, is commonly evaluated using the Go/NoGo task. While previous research has demonstrated that short-term practice can influence both behavioral and neural markers of response inhibition, the role of task difficulty—particularly when manipulated through short interstimulus intervals (ISIs)—remains underexplored. This study investigated the effects of short-term repeated practice on behavioral performance and neural activity during a high-difficulty Go/NoGo task with a short ISI. Methods: Fifteen healthy young adults completed a visual Go/NoGo task in four repeated sessions within a single day. The task involved a 600 ms ISI, 100 ms stimulus duration, and a 20% NoGo stimulus frequency. Behavioral outcomes included response time (RT) and error rate (ER). Neural activity was recorded via electroencephalography (EEG), focusing on event-related potentials (ERPs) associated with response inhibition, specifically the NoGo-N2 and NoGo-P3 components. Results: No significant changes were observed in RT or ER across sessions, indicating no improvement in behavioral performance. Similarly, NoGo-N2 amplitudes remained stable. However, a significant reduction in NoGo-P3 amplitude at the Fz electrode was found in later sessions, suggesting decreased frontal cortical engagement in response inhibition. Conclusions: Although short-term repeated practice of a high-difficulty Go/NoGo task did not enhance behavioral performance, it was associated with reduced neural activity related to response inhibition. These findings suggest that neurophysiological adaptations may occur even in the absence of observable behavioral changes, particularly under high task demands.

## 1. Introduction

In both daily life and sports activities, motor execution is often guided by external cues such as visual, auditory, and somatosensory stimuli. Response inhibition, a core aspect of executive function, refers to the ability to suppress planned or ongoing actions and plays a critical role in adaptive behavior [1,2]. Stimulus discrimination tasks—requiring individuals to either initiate or inhibit motor responses based on external stimuli—are widely used to investigate human sensorimotor function. Previous studies have demonstrated that older adults exhibit significantly longer reaction times (RTs) [3,4] and higher error rates (ERs) [5] in such tasks compared to younger, healthy individuals. Similarly, individuals with Parkinson’s disease (PD) have been reported to show impaired cognitive performance, with slower RTs and higher ERs than healthy controls [6,7].

Stimulus discrimination paradigms are commonly used to assess inhibitory control, wherein participants must decide whether to execute or inhibit a motor response based on external stimuli [8]. Among these, the Go/NoGo task is one of the most widely employed. In this task, participants are instructed to respond to a designated “Go” stimulus and to withhold their response to a “NoGo” stimulus. By presenting these stimuli in a randomized sequence, the Go/NoGo task enables the investigation of both motor execution and inhibition processes [9,10]. Previous studies have used RTs to Go stimuli and ERs to NoGo stimuli as behavioral indicators of inhibitory control [11]. Additionally, neural activity related to motor execution and inhibition has been examined using event-related potentials (ERPs) derived from electroencephalographic (EEG) recordings during the Go/NoGo task [12,13]. Among ERP components, a negative potential occurring approximately 200 ms after NoGo stimuli and a positive potential around 300 ms post-stimulus are considered key markers of inhibitory control, as their amplitudes are typically greater than those elicited by Go stimuli at similar latencies [10,14,15]. These components are referred to as N2 (Go-N2, NoGo-N2) and P3 (Go-P3, NoGo-P3), with NoGo-N2 and NoGo-P3 specifically regarded as neurophysiological indicators of inhibitory control [16,17].

Practice-related changes in inhibitory control have also been widely investigated. Previous research has demonstrated that engaging in cognitive tasks can induce changes in both brain activity and behavioral performance [18]. Similar effects have been observed in tasks targeting inhibitory control [19,20]. For instance, Kida et al. (2005) examined the impact of baseball batting practice on RTs using both simple reaction and Go/NoGo tasks, finding significant RT reductions only in the latter [21]. Yamashiro et al. (2015) similarly reported faster RTs in baseball players compared to non-players during a Go/NoGo task, with performance positively correlated with NoGo-N2 and NoGo-P3 amplitudes [22]. These results suggest that sport-specific training enhances inhibitory control and is associated with neuroplastic changes in the brain [23,24]. Other studies have explored the effects of short-term practice on inhibitory control. Manuel et al. (2010) reported that 40 min of Go/NoGo task practice reduced RTs but increased ERs [25], whereas Benikos et al. (2013) found improvements in both RTs and ERs with the same amount of practice [26]. These discrepancies indicate that the outcomes of practice may depend heavily on specific task parameters and conditions. Consequently, while the literature supports the idea that inhibitory control can be modified through practice, the consistency and nature of these effects remain inconclusive.

This variability is believed to stem from differences in task difficulty, which are influenced by variations in stimulus modality and task parameters. However, the optimal task conditions for enhancing inhibitory control through practice remain unclear. Task parameters include several components, such as interstimulus interval (ISI), stimulus probability, and stimulus presentation duration. Young et al. (2018) reported that shorter ISIs led to increased ERs [27]. Similarly, Nakata et al. (2005) found that manipulating ISI affected both RTs and ERPs latencies [28]. These findings indicate that both Go/NoGo task performance and the associated neural activity are influenced by task parameters. In particular, a short ISI is thought to increase demands on both response activation and inhibitory control, suggesting that different ISI conditions may engage distinct but interacting cognitive processes [29]. Therefore, variations in ISI may influence the extent and nature of practice effects observed in response inhibition tasks. Although previous studies on practice effects have primarily focused on transfer effects and changes in cognitive processing strategies, few have specifically examined how task parameters—particularly ISI—modulate practice-induced changes in inhibitory control.

In most previous studies, ISI has typically been set between approximately 1 and 3 s [25,26,30,31,32]. However, longer ISI are thought to reduce the demand for inhibitory control, as the extended temporal window allows for more deliberate decision-making processes [29]. Therefore, employing a shorter ISI is considered important for effectively investigating inhibitory control. To the best of our knowledge, no prior studies have examined the practice effects of inhibitory control tasks using an ISI shorter than 1 s. Accordingly, the present study aimed to investigate changes in inhibitory control following short-term repeated practice of a Go/NoGo task with a short ISI. To date, studies have investigated whether the practice effects of response inhibition tasks can be applied to the rehabilitation of disorders involving impaired inhibition, such as attention-deficit/hyperactivity disorder (ADHD) and binge eating disorder [32,33,34,35]. The findings of the present study may contribute to identifying appropriate task settings for rehabilitation and to further elucidating the neural mechanisms underlying response inhibition.

In this study, we hypothesized that short-term repeated practice of a Go/NoGo task would lead to improved performance, as indicated by shorter RTs and lower ERs—both behavioral indicators of inhibitory control. Additionally, previous studies have reported that repeated engagement of the inhibitory control network—which includes multiple brain regions involved in response inhibition—can lead to decreased activity not only in regions unrelated to inhibition, but also within the cortical areas directly associated with inhibitory control itself [20,36,37]. Therefore, we further hypothesized that the amplitudes of the NoGo-N2 and NoGo-P3 components, which are neural markers of inhibitory control, would decrease as practice duration increased.

## 2. Materials and Methods

### 2.1. Participants

Participants were young, healthy, right-handed adults who provided written informed consent after receiving both verbal and written explanations of the study procedures. The required sample size was calculated using G*Power (ver. 3.1.9.7), with a significance level (α) of 0.05 and a statistical power (1 − β) of 0.80. Based on a previous study that reported a partial η^2^ of 0.129, the corresponding effect size was estimated to be approximately 0.38 [38], indicating a minimum required sample size of 11 participants. Accordingly, 19 young, healthy, right-handed adults (11 men and 8 women) were initially recruited. After data screening, participants with fewer than 100 valid trials were excluded. As a result, the final sample comprised 15 participants (mean age: 22.9 ± 0.9 years; 9 men and 6 women). Exclusion criteria included a history of neurological or psychiatric disorders, alcohol or drug dependence, or the use of medications that affect the central nervous system. Additionally, participants were excluded from analysis if fewer than 100 valid trials remained due to error responses or EEG artifacts exceeding 100 µV, such as those caused by eye movements or body motion [39].

### 2.2. Experimental Procedures

**Figure 1:**
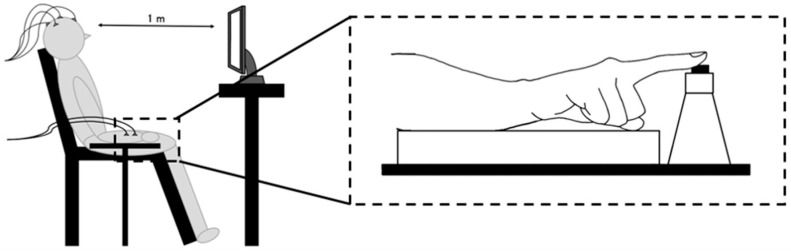
Experimental setup, including participant posture and stimulus presentation environment. Participants were seated comfortably in a reclining chair, with their right upper limb resting on a table positioned to the right of the chair. The right index finger was placed on a response button. Visual stimuli were presented on a 23.8-inch monitor located approximately 1 m in front of the participants.

Participants were comfortably seated in a reclining chair, with their right upper limb resting on a table positioned adjacent to the right side of the chair, and the forearm maintained in approximately 80° of pronation. In this position, the right index finger rested on a response button located on the table. A monitor displaying the visual stimuli was positioned approximately 1 m in front of the participant (Figure 1). The Go/NoGo task was administered across four trials, each consisting of four sets. Task performance metrics—RT and ER—as well as ERP components, were calculated for each trial. To reduce fatigue, rest intervals of approximately 1 min were provided between sets, and 5–10 min breaks were given between trials [40]. The total duration of the experiment, including EEG preparation, was approximately 2 h.

**Figure 2:**
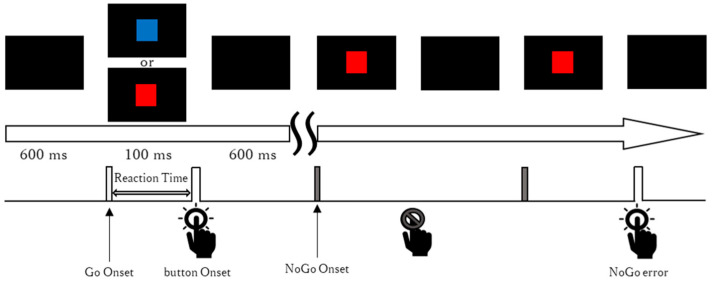
The Go/NoGo task procedure. Blue squares (Go stimuli) and red squares (NoGo stimuli) were presented for 100 ms, with a 600 ms ISI. RTs were measured from the onset of the Go stimulus to the button press. Responses to NoGo stimuli were recorded as NoGo errors.

A visual Go/NoGo task was used as the experimental paradigm. Visual stimuli were presented on a monitor with a 60 Hz refresh rate, controlled by Presentation software (ver. 23.1) (Neurobehavioral Systems, Berkeley, CA, USA) running on a personal computer. Participants were instructed to respond as quickly as possible to Go stimuli (blue squares) by pressing a button with their right index finger, and to withhold responses to NoGo stimuli (red squares). Each stimulus measured approximately 6 × 6 cm, corresponding to a visual angle of approximately 3.44°. Go/NoGo stimuli were presented for 100 ms, followed by a black screen during an ISI of 600 ms before the next stimulus appeared (Figure 2).

Go stimuli were presented with a probability of 80%, and NoGo stimuli with a probability of 20%, in a randomized order. Each set consisted of 200 trials, including 160 Go and 40 NoGo stimuli.

### 2.3. Data Recordings

#### 2.3.1. EEG Data

EEG data were recorded using a digital EEG system (Polymate Pro; Miyuki Giken, Tokyo, Japan). Electrodes were placed at nine scalp locations (F3, F4, C3, C4, P3, P4, Fz, Cz, Pz) according to the international 10–20 system [28,41], and signals were referenced to electrodes attached to both earlobes (A1, A2). To eliminate artifacts caused by eye blinks, electrooculograms (EOG) were recorded simultaneously from electrodes placed below and lateral to the right eye, along with the EEG signals [21]. EEG and EOG signals were sampled at 2000 Hz, with a band-pass filter of 0.03–200 Hz applied. Electrode impedance was maintained below 5 kΩ.

#### 2.3.2. Go/NoGo Stimuli and Button Responses

Go/NoGo stimuli were generated using Presentation software and delivered to the Polymate Pro as rectangular pulses. Button responses were recorded via a push button (Panasonic, Tokyo, Japan) directly connected to the Polymate Pro. To record muscle activity associated with button presses, surface electrodes were attached over the right flexor digitorum superficialis, and EMG signals were recorded in synchrony with the EEG.

### 2.4. Data Analysis

EEG and EMG data were analyzed using EMSE software (Version 5.6.1; Miyuki Giken, Tokyo, Japan). The following analyses were performed for each measurement variable.

#### 2.4.1. EEG Data

A band-pass filter of 0.1–60 Hz and a 50 Hz notch filter were applied. Analysis epochs were defined from −100 ms to 600 ms relative to the onset of the NoGo stimulus (0 ms), and ERPs were calculated by averaging across trials [42]. Trials containing artifacts, such as eye blinks within the analysis epoch, were excluded [21]. Amplitudes of the target components were calculated using the baseline-to-peak method. The baseline was defined as the mean voltage from −100 to 0 ms. The N2 peak was identified as the most negative deflection within 200–300 ms, and the P3 peak as the most positive deflection within 300–500 ms [43].

#### 2.4.2. Behavioral Data

RTs were calculated as the interval between the onset of the Go stimulus and the initiation of the button press, averaged across trials. ERs were calculated as the number of incorrect buttons pressed during NoGo trials divided by the total number of NoGo stimuli presented.

### 2.5. Statistical Analysis

Normality of the EEG and behavioral data was assessed using the Shapiro–Wilk test. As normality was confirmed, a one-way repeated-measures ANOVA was conducted with time (Trial 1, Trial 2, Trial 3, Trial 4) as a within-subject factor. For significant main effects, post hoc comparisons were performed using the Bonferroni method. All statistical analyses were performed using IBM SPSS Statistics version 25 (IBM Corp., New York, NY, USA), with the significance level set at 0.05.

## 3. Results

### 3.1. Task Performance

**Figure 3:**
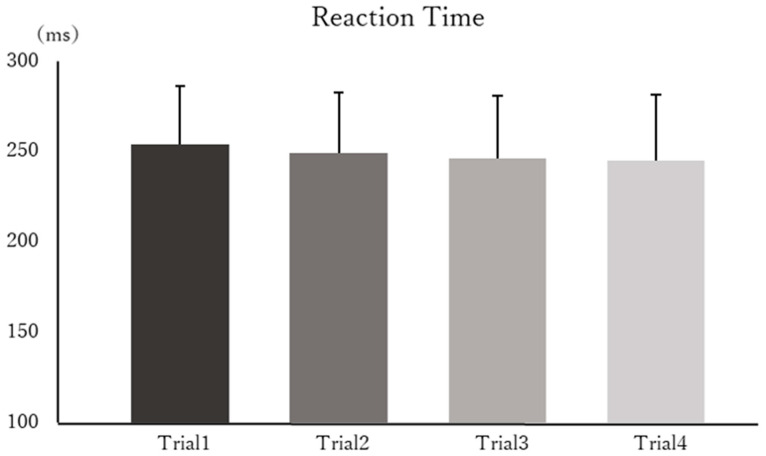
Mean reaction times (RTs) for each trial. The mean RTs (±standard deviation) across all participants is shown. No significant main effect of time was observed across trials.

**Figure 4:**
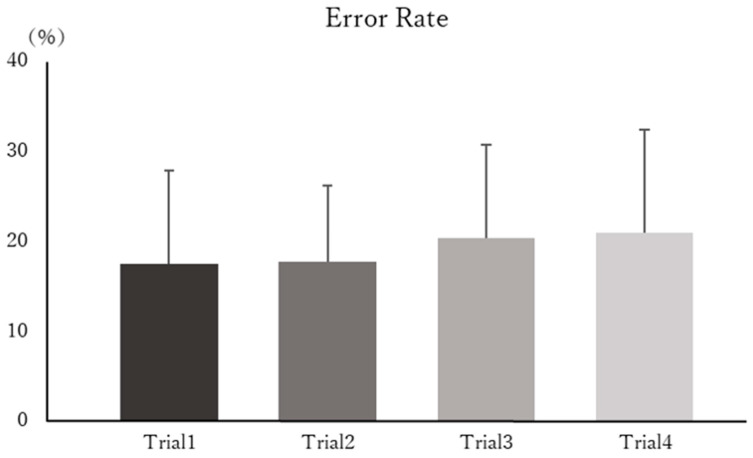
Mean error rates (ERs) for each trial. The mean ERs (±standard deviation) across all participants are shown. No significant main effect of time was observed across trials.

Figure 3 shows the mean RTs for each trial across all participants, and Figure 4 presents the mean ERs for each trial. Statistical analysis revealed no significant main effect of time on RTs (F(3, 39) = 1.641, *p* = 0.194, η^2^_p_ = 0.109). Similarly, no significant main effect of time was observed for ERs (F(3, 39) = 1.370, *p* = 0.265, η^2^_p_ = 0.080).

### 3.2. EEG Data

**Figure 5:**
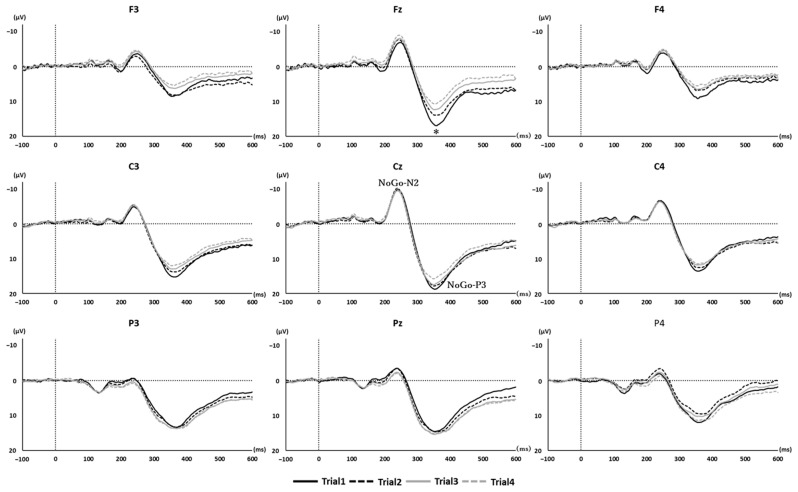
The grand average waveforms of NoGo-ERPs. The vertical axis represents amplitude (µV), and the horizontal axis represents time (ms). NoGo-N2 and NoGo-P3 are characterized by negative and positive deflections occurring approximately 200 ms and 300 ms, respectively, after the presentation of a NoGo stimulus, and they are considered to reflect response inhibition. The solid black line shows the NoGo ERPs waveform for Trial 1, the dotted black line for Trial 2, the solid gray line for Trial 3, and the dotted gray line for Trial 4. At Fz, the NoGo-P3 amplitude was significantly reduced in Trials 3 and 4 compared to Trial 1. An asterisk (*) denotes a significant main effect of time (*p* < 0.05).

Figure 5 displays the grand average waveforms for NoGo trials across all participants. Table 1 and Table 2 present the mean amplitudes of the NoGo-N2 and NoGo-P3 components for each trial.

Statistical analysis showed no significant main effects of time on NoGo-N2 amplitudes at any recording site (F3, F(3, 39) = 3.109, *p* = 0.061, η^2^_p_ = 0.161; F4, F(3, 39) = 0.754, *p* = 0.527, η^2^_p_ = 0.053; C3, F(3, 36) = 0.131, *p* = 0.846, η^2^_p_ = 0.012; C4, F(3, 39) = 0.862, *p* = 0.438, η^2^_p_ = 0.078; P3, F(3, 39) = 0.417, *p* = 0.742, η^2^_p_ = 0.026; P4, F(3, 39) = 1.119, *p* = 0.353, η^2^_p_ = 0.079; Fz, F(3, 39) = 1.258, *p* = 0.298, η^2^_p_ = 0.087; Cz, F(3, 36) = 0.204, *p* = 0.792, η^2^_p_ = 0.018; Pz, F(3, 36) = 1.303, *p* = 0.288, η^2^_p_ = 0.101).

A significant main effect for NoGo-P3 amlitudes was found only at Fz (F(3, 39) = 5.210, *p* = 0.004, η^2^_p_ = 0.271). No significant effects were observed at the other sites (F3, F(3, 39) = 2.288, *p* = 0.094, η^2^_p_ = 0.139; F4, F(3, 39) = 1.395, *p* = 0.259, η^2^_p_ = 0.112; C3, F(3, 39) = 2.590, *p* = 0.067, η^2^_p_ = 0.117; C4, F(3, 39) = 0.936, *p* = 0.433, η^2^_p_ = 0.052; P3, F(3, 39) = 1.353, *p* = 0.275, η^2^_p_ = 0.087; P4, F(3, 39) = 2.518, *p* = 0.072, η^2^_p_ = 0.145; Cz, F(3, 39) = 0.861, *p* = 0.433, η^2^_p_ = 0.065; Pz, F(3, 39) = 1.484, *p* = 0.247, η^2^_p_ = 0.085). Post hoc analysis for Fz revealed that the NoGo-P3 amplitude was significantly reduced in Trial 3 (*p* = 0.047) and Trial 4 (*p* = 0.045), compared with Trial 1.

## 4. Discussion

In this study, we investigated changes in response inhibition following short-term repeated practice of a Go/NoGo task with a short ISI. The results revealed no significant changes in task performance or in NoGo-N2 amplitudes. However, the NoGo-P3 amplitude at the Fz electrode decreased following short-term repeated practice.

### 4.1. Task Performance

Previous studies have reported that RTs reflect the interval from stimulus presentation to response selection [44,45], while ERs reflect the accuracy of stimulus-response selection [27]. Therefore, in response to inhibition tasks such as the Go/NoGo task, RTs and ERs have been widely used as behavioral indices of response inhibition [11]. Several studies have demonstrated that short-term practice of such tasks leads to reductions in both RTs and ERs [20,46,47]. However, no significant changes in RTs or ERs were observed in the present study. The effects of task practice have been shown to depend on factors such as task difficulty and the amount of practice, including its duration [26,48,49]. Regarding task difficulty, it has been reported that practice effects are less likely to occur under high-difficulty conditions [26], which are typically associated with slower RTs and higher ERs [26,50]. Based on these findings, we defined a high-difficulty task as one in which achieving superior performance—such as shorter RTs or lower ERs—is particularly challenging.

In Go/NoGo tasks, task difficulty has been reported to be influenced by factors such as ISI, stimulus presentation ratio, and stimulus duration [27,28,51]. Most previous studies employed an ISI between 1 and 3 s and found that shorter ISI reduces RTs but increases ERs [27,28], suggesting that shorter ISI increases task difficulty. Regarding stimulus presentation ratio, ERs tend to be higher when the NoGo stimulus ratio is 20% compared to 50% [27,28], indicating that a lower NoGo ratio increases difficulty. Furthermore, while stimulus durations of approximately 200 ms are commonly used [11,26,50], shorter durations have been shown to impair stimulus discrimination and increase ERs [51]. Thus, the present study—which employed an ISI of 600 ms, a NoGo stimulus ratio of 20%, and a stimulus duration of 100 ms—likely imposed a higher level of difficulty than previous studies. Additionally, Benikos et al. (2013) defined a high-difficulty task as one requiring a response within 300 ms of Go stimulus presentation, noting that such a brief window reduces response accuracy and induces a speed-accuracy trade-off [26,50]. The mean RTs in the present study were approximately 250 ms, likely reflecting the rapid response shifting required by the short ISI [29]. As in previous studies [26,50], this condition likely contributed to the task’s difficulty due to the speed-accuracy trade-off.

Indeed, the mean ERs in the present study were approximately 20.0%, which is slightly higher than the rates reported in previous studies [25,28,41,50,52,53]. Benikos et al. (2013) reported ERs of 7.4% for low-difficulty, 11.1% for medium-difficulty, and 25.0% for high-difficulty conditions in a Go/NoGo task [26], further supporting the notion that the task used in the present study was highly challenging, potentially reducing the likelihood of observing practice effects. Regarding practice duration, Manuel et al. (2010) reported that approximately 40 min of Go/NoGo task practice led to increased ER but reduced RTs [25]. Similarly, Benikos et al. (2013) found that a comparable training duration reduced RTs [26]. In the present study, practice duration was also approximately 40 min, suggesting that sufficient time was provided to elicit practice effects. However, due to the high difficulty, this duration may have been insufficient to produce observable behavioral improvements. In addition, the Go/NoGo task employed in the present study had a simple structure, in which two types of visual stimuli were presented at fixed intervals. Benikos et al. (2013) examined practice effects using a task in which two types of stimuli were presented at randomized intervals, which varied across measurement blocks [26,50]. Similarly, Manuel et al. (2010) also used a task in which stimuli were presented at random intervals [25]. Therefore, it is unlikely that the task structure used in this study was complex or that task difficulty was elevated. These findings suggest that the Go/NoGo task with a short ISI imposes a high level of difficulty on participants, and that short-term practice alone may not be sufficient to yield measurable improvements in task performance.

### 4.2. EEG Data

Among the ERPs derived from EEG recordings during the task, the N2 component is thought to reflect pre-response stimulus discrimination processes [54]. Specifically, the NoGo-N2 amplitude has been reported to reflect the strength of response inhibition [51,55,56,57], although other studies suggest that it reflects response conflict [17,58]. Response conflict refers to the competition during response selection—specifically, between execution and inhibition—in stimulus discrimination tasks [17,58]. In the Go/NoGo task, participants are required to respond to a designated “Go” stimulus and to withhold responses to a “NoGo” stimulus, thereby inducing response conflict through the continuous need to choose between pressing or not pressing the button [17]. Previous studies have reported that NoGo-N2 amplitude increases when the NoGo stimulus ratio is 20% compared to 50%, suggesting that stronger response conflict occurs under conditions with lower NoGo frequency [59]. Similarly, the short ISI used in this study may have provided insufficient time for response selection, thereby enhancing response conflict. Therefore, it is likely that the Go/NoGo task employed in this study induced strong response conflict due to both the low NoGo stimulus ratio and the short ISI.

Previous studies have reported that short-term repeated practice, similar to that employed in the present study, does not alter NoGo-N2 amplitude [60]. For example, Benikos et al. (2013) found that approximately 40 min of practice on a response inhibition task did not affect NoGo-N2 amplitude [26]. In contrast, Schapkin et al. (2007) reported that NoGo-N2 amplitude was modulated following 20 min of daily practice over three consecutive days [61]. These findings suggest that NoGo-N2 amplitude is unlikely to change in response to short-term practice conducted within a single day. Therefore, in the present study, it is considered that the task involved strong response conflict, and as a result, neural activity related to response conflict was not reduced, leading to no decrease in NoGo-N2 amplitude following short-term repeated practice.

In this study, only the NoGo-P3 amplitude at the Fz electrode decreased following short-term repeated practice. Previous studies have reported that individuals with lower ER exhibit larger NoGo-P3 amplitudes compared to those with higher ERs [62], suggesting that NoGo-P3 amplitude reflects the strength of response inhibition [16]. However, repeated practice of response inhibition tasks has also been associated with reduced neural activity as inhibitory control improves [20,36,37]. Specifically, cortical activity has been shown to decrease with repeated stimulus presentation [60,63], and Hartmann et al. (2016) reported that short-term repeated practice reduces cortical activation in response inhibition tasks [47]. Furthermore, Zhang et al. (2015) found that expert fencers—who are considered to possess enhanced inhibitory control—exhibited superior task performance but smaller NoGo-P3 amplitudes compared to non-experts [64]. These findings suggest that repeated practice involving inhibitory control—whether through experimental tasks or sport-specific training—may promote more efficient processing, resulting in reduced neural activity.

A reduction in the NoGo-P3 amplitude was observed exclusively at the frontal electrode site Fz in the present study. The P3 component is typically recorded from midline electrodes (Fz, Cz, Pz) and is thought to reflect the allocation of attentional resources, particularly in response to low-frequency stimuli [39,65,66,67,68]. Since NoGo stimuli were presented infrequently, it is possible that the observed P3 reduction was related to changes in attentional allocation. However, studies using repeated oddball paradigms have demonstrated that P3 amplitudes elicited by rare stimuli generally remain stable over time [69]. In addition, P3 amplitude is considered a marker of cognitive load during task performance. Prior research has shown that P3 amplitude decreases as task difficulty and cognitive demands increase [50,70,71]. Such reductions, however, have mainly been observed in the parietal region and are thought to reflect processes distinct from those associated with changes in frontal P3 activity [50]. Moreover, given that participants performed the same task repeatedly in this study, it is unlikely that cognitive load increased as the task progressed. Therefore, the observed reduction in NoGo-P3 amplitude at Fz cannot be fully attributed to either reduced attentional allocation or elevated cognitive load. Response inhibition engages a network of brain regions, particularly within the prefrontal cortex—including the inferior frontal gyrus, pre-supplementary motor area, and anterior cingulate cortex [72,73,74,75]. The NoGo-P3 component is believed to originate in the prefrontal cortex and anterior cingulate cortex, both of which are critical to inhibitory control [67,76,77,78]. Previous studies have shown that neural activity in these frontal areas, such as the inferior frontal gyrus, diminishes with repeated practice of response inhibition tasks [36,47]. Accordingly, the reduced NoGo-P3 amplitude at Fz likely reflects decreased activation within the inhibitory control network.

Importantly, no changes in task performance were observed with repeated practice, suggesting that behavioral improvement in inhibitory control did not occur. Prior studies have indicated that neurophysiological adaptation may precede behavioral changes in stimulus discrimination tasks [79], implying that brain-level modifications can emerge independently of overt performance improvements. While prolonged task performance has been linked to fatigue-related decreases in NoGo-P3 amplitude [40], such fatigue is typically accompanied by impaired task performance, which was not evident in this study. Therefore, it is unlikely that fatigue significantly influenced the observed neural changes. In summary, short-term repeated practice of a response inhibition task with a short ISI does not lead to measurable behavioral improvements but may reduce frontal neural activity associated with inhibitory processing.

### 4.3. Limitation

This study employed visual stimuli in the Go/NoGo task. Previous research has demonstrated that neural responses to NoGo stimuli vary depending on the sensory modality [16,52]. Therefore, a key limitation of the present study is that it did not examine whether neurophysiological responses associated with response inhibition differ across modalities. Future studies should investigate whether similar neural adaptations occur when using auditory or somatosensory stimuli. Additionally, the present study focused solely on the short-term effects of repeated practice conducted within a single day. As such, it was not possible to evaluate the long-term retention or durability of the observed neural changes. Moreover, the brief duration of practice may have been insufficient to produce measurable behavioral or neurophysiological adaptations. Future research should employ extended training protocols to more thoroughly assess the long-term effects of repeated practice on inhibitory control. In addition, although efforts were made to minimize fatigue during task practice, it was not possible to completely eliminate its influence. Therefore, the results of this study should be interpreted with caution.

## 5. Conclusions

The present study demonstrated that a Go/NoGo task with a short ISI imposed a high level of cognitive demand, and as a result, short-term repeated practice did not lead to improvements in task performance. However, it also revealed that such practice reduced neural activity associated with response inhibition, independent of behavioral changes. These findings suggest that neurophysiological adaptations can occur even in the absence of measurable behavioral improvements, highlighting a potential dissociation between neural and behavioral responses to repeated practice.

## Figures and Tables

**Table 1:** Mean NoGo-N2 amplitudes for each trial and electrode.

	F3	F4	Fz	C3	C4	Cz	P3	P4	Pz
Trial 1	−4.82 (4.01)	−5.43 (6.85)	−7.58 (5.18)	−6.10 (7.70)	−7.89 (10.57)	−11.51 (6.08)	−0.66 (5.29)	−3.15 (6.27)	−5.26 (6.57)
Trial 2	−3.10 (4.55)	−4.27 (7.59)	−6.45 (5.65)	−5.70 (6.25)	−5.74 (8.08)	−9.03 (5.58)	−0.53 (5.41)	−3.51 (4.88)	−4.13 (3.25)
Trial 3	−5.38 (3.71)	−5.52 (5.79)	−8.27 (3.21)	−6.39 (3.56)	−7.28 (5.73)	−10.55 (3.18)	−1.03 (3.62)	−2.94 (4.48)	−3.23 (3.48)
Trial 4	−5.56 (6.43)	−6.05 (4.80)	−9.06 (3.82)	−6.18 (2.85)	−7.26 (5.09)	−10.90 (3.05)	0.10 (4.58)	−1.81 (4.19)	−2.82 (4.79)

Data are expressed as mean (standard deviation).

**Table 2:** Mean NoGo-P3 amplitudes for each trial and electrode.

	F3	F4	Fz	C3	C4	Cz	P3	P4	Pz
Trial 1	9.05 (6.70)	9.46 (8.59)	16.35 (7.61) *	15.48 (7.70)	14.11 (10.57)	19.53 (6.08)	14.80 (5.29)	13.18 (6.27)	15.80 (6.57)
Trial 2	8.54 (5.80)	7.41 (8.38)	13.02 (5.87)	13.49 (6.87)	12.35 (9.33)	16.99 (6.94)	12.94 (5.55)	9.59 (6.83)	14.27 (6.39)
Trial 3	7.64 (5.67)	7.74 (7.34)	12.96 (5.53) *	13.94 (6.22)	12.80 (7.01)	18.40 (4.62)	14.91 (6.41)	11.74 (6.51)	16.75 (5.79)
Trial 4	6.43 (5.36)	6.87 (7.07)	11.81 (5.62) *	13.14 (4.95)	12.69 (6.16)	17.19 (4.28)	14.91 (6.28)	12.58 (6.55)	16.75 (5.52)

Data are expressed as mean (standard deviation). An asterisk (*) denotes a significant main effect of time (*p* < 0.05).

## Data Availability

The data presented in this study is available on request from the corresponding author. The data is not publicly available due to privacy reasons.
